# Snot what you think: Mucus or myxoid matrix with epithelioid cells and bubbly cytoplasm?

**DOI:** 10.1111/cyt.13036

**Published:** 2021-10-12

**Authors:** Kathrin Ludwig, Rachele Biancotti, Lara Alessandrini, Ambrogio Fassina

**Affiliations:** ^1^ Padua University Hospital Padova Italy; ^2^ University of Padova Padova Italy

## Abstract

A case of progressive nasal obstruction in a 63 year old man is described. FNA cytology yielded dominant myxoid matrix with charateristic epithelioid cells, round nuclei with nucleoli, and eosinophilic, granular or vacuolated cytoplasm to allow a diagnosis tto be made.

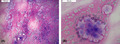

## 1 CASE HISTORY

A 63‐year‐old man presented with an 18‐month history of progressive nasal obstruction, episodes of catarrhal otosalpingitis, and rare episodes of epistaxis.

An MRI scan of the craniofacial structures showed an expansive, lobular lesion of the nasopharynx measuring 37 × 23 × 44 mm, invading the sphenoid sinus, focally eroding the clivus and extending posterolaterally in proximity of, but not involving, the foramen lacerum.

The patient had no previous history of tumours.

**FIGURE 1:**
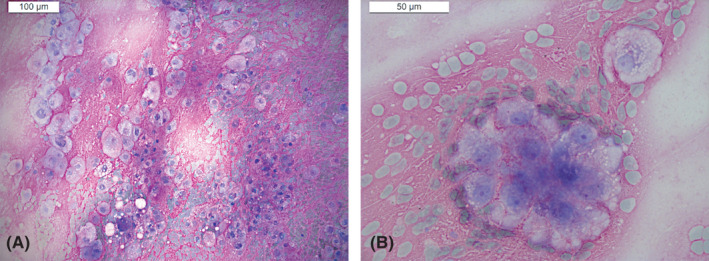
Fine needle aspiration cytology shows a dominant myxoid matrix with numerous embedded epithelioid cells with round nuclei, rare small nucleoli, and eosinophilic granular or abundantly vacuolated cytoplasm (A), focally in small aggregates (B)

Endoscopic‐guided fine needle aspiration cytology yielded moderately cellular smears of epithelioid cells with round nuclei, evident nucleoli, and eosinophilic granular or abundantly vacuolated cytoplasm, embedded in a dominant myxoid matrix (Figure 1).

## 2 MORPHOLOGY QUIZ

1. Which of the following is the most likely diagnosis based on clinical information and cytology (Figure 1)?
  A Olfactory neuroblastoma
  B Chordoma
  C Extraskeletal myxoid chondrosarcoma
  D Myoepithelial carcinoma
  E Malignant peripheral nerve sheath tumour.

**FIGURE 2:**
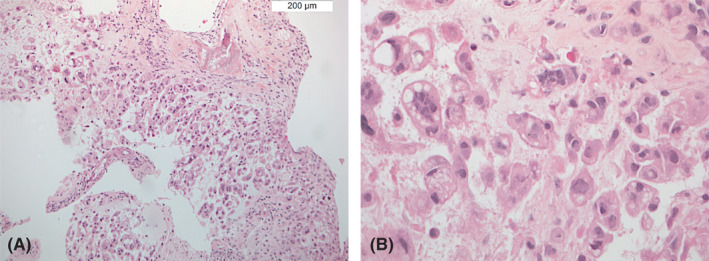
Histopathological sections (H&E) show a proliferation of epithelioid neoplastic cells, arranged in lobules, nests, and anastomosing cords, with abundant eosinophilic (A) and, occasionally, bubbly/vacuolated cytoplasm (B); nuclei are predominantly hyperchromatic, with low nuclear‐to‐cytoplasmic ratio and minimal atypia

After an initial biopsy, which turned out to be non‐diagnostic, the patient underwent endoscopic type II nasopharyngectomy. Histopathological examination of the surgical specimen on haematoxylin and eosin (H&E) stained slides revealed a proliferation of epithelioid neoplastic cells, arranged in lobules and nests, with abundant clear to eosinophilic and, occasionally, bubbly/vacuolated cytoplasm. The nuclei were hyperchromatic, with low nuclear‐to‐cytoplasmic ratio and minimal atypia (Figure 2).

2. Which of the following stains would aid you in the differential diagnosis?
  A CD 56
  B Calretinin
  C D2‐40
  D Glial fibrillary acidic protein (GFAP)
  E Brachyury.
3. What is the name of the characteristic epithelioid cells with their eosinophilic granular or abundant and vacuolated cytoplasm?
  A Cartwheel cells
  B Odontoblasts
  C Langerhans cells
  D Physaliferous cells
  E Ganglion cells.
4. Which of the following tumours does not enter in the differential diagnosis?
  A Chondrosarcoma
  B Chordoid meningioma
  C Glioma
  D Clear cell renal carcinoma
  E Merkel cell carcinoma.

**FIGURE 3:**
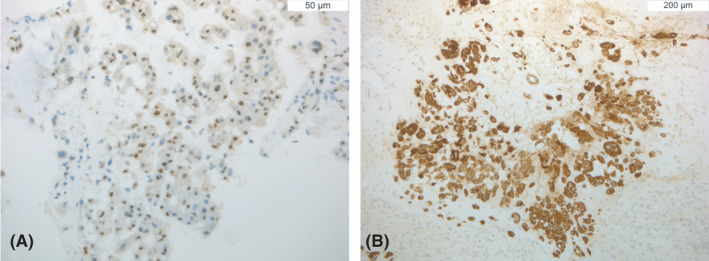
Immunohistochemical analysis displaying positive nuclear immunostaining for brachyury (A) and a positive membranous and cytoplasmic staining for MNF‐116 (B)

Immunohistochemical analysis of neoplastic cells revealed a positive nuclear immunostaining for brachyury and a positive membranous staining for pan‐cytokeratins (Figure 3) as well as EMA, while S100 staining was negative.

**ANSWERS TO MORPHOLOGY QUIZ**

**QUESTION** **1**

Answer: B

Chordoma, a neoplasm of notochordal differentiation, is a rare subtype of bone sarcoma typically occurring along the axial skeleton and mainly involving the following locations in decreasing order of frequency: mobile spine (32.8%), skull‐base (32%), and sacrum and coccyx (29.2%).^1^ Extraskeletal cases have been anecdotally described, but are exceedingly rare.^1^

When encountered in the head and neck region, chordomas are mostly located in the skull base, with only a minority of cases being located in the cervical spine. However, cases in extra‐axial locations of the head and neck have been reported and include the soft tissue of the cervix, the oro‐ and nasopharynx, the paranasal sinuses, and the lateral nasal wall.^2^

According to the most recent WHO classification of soft tissue and bone tumours three different types of chordoma exist, namely the conventional chordoma (including the chondroid subtype), the dedifferentiated chordoma, and the poorly differentiated chordoma.^1^

Chordoma incidence has been shown to display a strong age‐related variability, being unequivocally low in the paediatric age group and peaking in the eighth decade; its overall incidence has been reported to be approximately 0.08/100,000 in the United States and Europe with a median age of about 60 years at diagnosis.^3^

However, the poorly differentiated variant, characterised by the loss of SMARCB1 expression, is mainly diagnosed in the paediatric population.

Clinically, conventional chordoma is a slow‐growing tumour with a strong tendency towards local recurrence, and more that 40% of those arising in sites other than the skull base metastasise to the lung, bone, lymph nodes, and subcutaneous tissue.^1^ Symptoms are often related to mass effect, including pain, site‐related neurological symptoms, and in cases of head and neck localisation, headache, diplopia, and cranial nerve palsy. Dedifferentiated and poorly differentiated chordomas show symptoms similar to those of conventional chordomas; however, progression tends to be more rapid with a high mortality and metastatic rate.^1^

Macroscopically, conventional chordomas appear as solid, lobulated masses, with a variably gelatinous or chondroid cut surface and areas of haemorrhage, while dedifferentiated chordomas show a more biphasic appearance in which the dedifferentiated component is that of a solid high‐grade sarcoma.^1^

Microscopically, conventional chordoma is composed of large epithelioid cells with clear to light eosinophilic cytoplasm, arranged in cords and nests and separated into lobules by fibrous septa. Substantial intratumoural cytologic heterogeneity can be observed. The abundant extracellular matrix in which the cells are embedded can mimic the background of hyaline cartilaginous tumours, thus, when prominent, conferring the name of “chondroid chordoma” to a subtype of conventional chordomas. Dedifferentiated and poorly differentiated chordomas differ from conventional ones in that they display a simultaneous component of high‐grade spindle and/or pleomorphic sarcoma and rhabdomyoid differentiation of the epithelioid cells with no/scarce myxoid matrix, respectively.

Cytologically, conventional chordomas display variably cellular smears, characterised by large epithelioid round to oval cells, discohesive or in solid aggregates, with clear to light eosinophilic vacuolated cytoplasm, immersed in a frequently dominant extracellular myxoid background. Chordoma can show a substantial degree of inter‐ and intratumoural cytological heterogeneity, with nuclear atypia and pleomorphism, ranging from low to (less commonly) severe. In the latter case, signs of necrosis might be present.

Although chordomas are regarded as low‐grade, slowly growing tumours they have a high local recurrence rate and significant mortality with a median survival of 7 years,^1^ and a 5‐year overall age‐adjusted relative survival of 72% and 61% in the United States and Europe, respectively.^3^

Primary treatment options for chordoma patients consist of radical surgical resection. Chemotherapy or radiotherapy are performed only in conventional and poorly differentiated variants, whereas the dedifferentiated variant shows only negligible benefits and surgery is the only option.

**QUESTION** **2**

Answer: E

Ancillary studies, including immunohistochemistry, are frequently required in the differential diagnosis of chordoma and its mimics. The most discriminatory immunohistochemical markers are combined cytokeratin and brachyury, both expressed in conventional chordomas.

Brachyury, a transcriptional factor known to be crucial in notochord development, is considered to be the diagnostic hallmark for conventional chordoma. Furthermore, conventional chordoma displays diffuse immunoreactivity for EMA, and variable S100 positivity, while expression of both markers is typically lost in the dedifferentiated variant.

Nuclear brachyury positivity is retained in the poorly differentiated variant, while it is lost in the dedifferentiated one. INI‐1 expression, while retained in conventional chordoma, is constantly lost in the poorly differentiated variant as well as in a subset of dedifferentiated chordomas.

**QUESTION** **3**

Answer: D

The diagnostic physaliferous (from Greek *physallis* [“bubble”] and *pheros* [“bearing”]) cells have abundant eosinophilic cytoplasm and variable intracytoplasmic vacuoles.

**QUESTION** **4**

Answer: E

The differential diagnosis of chordoma comprises several lesions, including conventional chondrosarcoma, extraskeletal myxoid chondrosarcoma, meningioma and glioma (especially the chordoid variants), metastatic adenocarcinoma (including renal clear cell carcinoma), and myoepithelial tumours/myoepithelial rich tumours.

The differential diagnosis of chordoma with the above‐mentioned tumour entities can be exceptionally challenging. However, the expression of brachyury (encoded by the *TBXT* gene), the hallmark of chordoma, should help to unravel the puzzle in most cases.

Chondrosarcoma, although challenging due to similar clinical, imaging, and histomorphological findings, is negative for brachyury expression; this is particularly important as some markers involved in chondrogenesis, such as D2‐40, SOX‐9, can be positive in both chondrosarcoma and in chordoma. Keratins are only very rarely expressed in conventional and extraskeletal myxoid chondrosarcoma. Due to the loss of brachyury expression in dedifferentiated chordomas, its differential diagnosis of dedifferentiated chondrosarcomas is particularly challenging.

Meningioma and glioma (including chordoid variants) can express cytokeratin, EMA and S100; however, brachyury expression is negative. Additional stains such as GFAP and PR will help in establishing the diagnosis.

Metastatic adenocarcinomas, including clear cell renal carcinoma, do not express brachyury. Molecular and cytogenetic investigations can further help to narrow down the differential diagnosis in cases in which immunohistochemistry has not proved resolutive. For example, identification of *NR4A3* gene rearrangements is helpful in the differential diagnosis with extraskeletal myxoid chondrosarcoma.

## CONFLICT OF INTEREST

The authors declare there are no conflicts of interest.

## AUTHOR CONTRIBUTIONS

All authors have contributed substantially to the manuscript by either writing it, tending to the photos, the cytological and histological diagnoses or by critically reviewing the literature and the manuscript.

## Data Availability

Data sharing is not applicable to this article as no new data were created or analysed in this study.
